# Neutrophil Extracellular Traps, Antiphospholipid Antibodies and Treatment

**DOI:** 10.3390/antib6010004

**Published:** 2017-03-06

**Authors:** Jessica Bravo-Barrera, Maria Kourilovitch, Claudio Galarza-Maldonado

**Affiliations:** 1 UNERA (Unit of Rheumatic and Autoimmune Diseases), Hospital Monte Sinaí, Miguel Cordero 6-111 y av. Solano, Cuenca, Ecuador; jessica.bravo@uneracuenca.com (J.B.-B.); claudiogalarza@hotmail.com (C.G.-M.); 2 Department of Hematology and Hemostasis, CDB, Hospital Clinic, Villaroel 170, 08036 Barcelona, Catalonia, Spain; 3 Faculty of Medicine and Health Science, Doctorate Programme “Medicine and Translational Research”, Barcelona University, Casanova, 143, 08036 Barcelona, Catalonia, Spain; 4 Department of Investigation (DIUC-Dirección de Investigación de Universidad de Cuenca), Cuenca State University, Av. 12 de Abril y Agustin Cueva, Cuenca, Ecuador; claudiogalarza@hotmail.com

**Keywords:** neutrophil extracellular traps, NETosis, autophagy, antibodies, antiphospholipid syndrome

## Abstract

Neutrophil extracellular traps (NETs) are a network of extracellular fibers, compounds of chromatin, neutrophil DNA and histones, which are covered with antimicrobial enzymes with granular components. Autophagy and the production of reactive oxygen species (ROS) by nicotinamide adenine dinucleotide phosphate (NADPH) oxidase are essential in the formation of NETs. There is increasing evidence that suggests that autoantibodies against beta-2-glycoprotein-1 (B2GP1) induce NETs and enhance thrombosis. Past research on new mechanisms of thrombosis formation in antiphospholipid syndrome (APS) has elucidated the pharmacokinetics of the most common medication in the treatment of the disease.

## 1. Introduction

Neutrophils are granulocytes that have an essential role in the pathology of a broad spectrum of inflammatory diseases. In circulation, the neutrophils remain inactive; but under inflammatory conditions, they are recruited to the tissues, where they participate in the destruction of pathogens through different mechanisms. The neutrophils’ activation occurs via a variety of receptors, including pattern-recognition receptors and Fc-receptors [1]. For decades, phagocytosis was considered the primary mechanism by which neutrophils targeted infections [2]. However, in 2004, Brinkmann et al. described another distinct antimicrobial activity of neutrophils, in which neutrophils were shown to release extracellular traps (NETs) [3]. Steinberg and Grinstein named this process of neutrophil cell death as “NETosis” [4].

NETs are a network of extracellular fibers, compounds of decondensed chromatin, including neutrophil DNA and high affinity histones, which are covered with antimicrobial enzymes and granular components, such as myeloperoxidase (MPO), neutrophil elastase (NE), cathepsin G and other microbicidal peptides [3,5].

In vitro studies, using the non-physiological stimulus phorbol-12-myristate-13-acetate (PMA), demonstrated that during NETs formation, a rupture of the cell membrane and exposure of the inner membrane phospholipids occur. NETosis was classified as a novel type of cell death [6]. However, there is an ongoing controversy on whether or not the death of neutrophils actually occurs in vivo. Through detailed observations of neutrophil behavior on Gram-positive skin infections in mice and humans, Yipp et al. were able to demonstrate that while neutrophils form and release NETs during crawling and become anuclear, they do not show any signs of programmed cell death [7].

Further studies are needed to elucidate whether or not anuclear neutrophils have the capacity to activate other cell mechanisms and functions [8]. 

The interest in the role of NETs in autoimmune diseases arose with the discovery of certain mechanisms that trigger NETosis by non-infectious stimuli, such as: immune complexes, autoantibodies, cytokines, cholesterol and monosodium urate (MSU) crystals [1]. Multiple studies have shown the implication of such mechanisms in NETs formation in chronic inflammatory processes, as seen in lung [9], systemic lupus erythematosus [10], antineutrophil cytoplasmic antibodies (ANCA)-associated vasculitis [11], rheumatoid arthritis [12], gouty arthritis [13,14], familiar Mediterranean fever [15], psoriasis [16] and autoimmune coagulation disorders [17,18].

In susceptible individuals, many of the molecules released through NETosis (for example, double-stranded (ds) DNA, histones, cytokines, MPO, etc.) could be recognized by the immune system as autoantigens and initiate the autoimmune response. If this occurs, a vicious cycle of autoimmune reactions is triggered, which leads to further release of antigenic material [19].

In this review, we will address the contribution of NETosis in the development of antiphospholipid-mediated pathology. Furthermore, we will identify NETosis-related aspects of the pharmacokinetics of medication used in the treatment of APS.

## 2. NETs Formation

During NETs formation, the neutrophils lose their variability, which results in the activation of certain signaling pathways producing the dissolution of the nuclear envelope [6]. Remijsen, et al. proved that autophagy and the production of reactive oxygen species (ROS) by NADPH-oxidase are essential in the formation of NETs [20]. The NADPH enzyme is activated in response to the threat of infection, triggering the generation of antimicrobial reactive oxidants [21]. The inhibition of either autophagy or NADPH-oxidase prevents decondensation of intracellular chromatin; without the ability to complete these processes, NETosis cannot occur [20,22].

ROS is a signaling molecule that can promote inflammation and tissue damage [23]. The generation of ROS is necessary for the activation of neutrophil enzymes, which produce DNA unwinding, a critical process in NETosis [24]. As NETosis is dependent on ROS production by NADPH-oxidase, the inability to form ROS in genetically-defective NADPH-oxidase patients prevents NETs formation [6,25].

Cytokines are activators of neutrophil functions and, consequently, play an important role in the process of NETosis. The neutrophils of healthy subjects, treated with TNF-α, IL-1β or IL-8, produce free radicals, and NETs form by the activation of NADPH-oxidase. This findings point out the importance of cytokines in the enhanced release of NETs in systemic inflammatory responses syndrome [26]. Cytokines, such as TNF-α, IL-1β, IL-8 and IL-6, have been observed to enhance free radical generation. Moreover, a variety of studies emphasize the significant role of TNF-α in mitochondrial ROS production [27,28].

It is important to note that aggregated NETs have been observed to regulate inflammation through the degradation of cytokines and chemokines, limiting the inflammation in patients with MSU deposits [29].

Platelets are one of the important actors in the immune response and play a critical role in NETs formation [30]. When platelets stimulation occurs, they begin to secrete molecules that can modulate the activation of neutrophils. One such molecule is high mobility group box 1 (HMGB1), a damage-associated molecular pattern molecule. HMGB1 is released as a result of cell death and is an important marker of inflammatory response to tissue damage. Recently, it has been demonstrated that the HMGB1-platelets complex is one of the key inductors of NETs formation. In addition, HMGB1 regulates cell death through the management of apoptosis, autophagy and necrosis in cells [31,32]. The capacity of HMGB1 to inhibit apoptosis can explain the absence of observed cell death in anuclear neutrophils following NETs in vivo*.*

### 2.1. Autophagy and NETosis

Autophagy was defined over 40 years ago by Christian de Duve as the “eating of self” [33], and through the work of Yoshinori Ohsumi (2016 Nobel Prize winner in physiology or medicine), the mechanisms and genes of autophagy have been elucidated [34,35]. 

Autophagy is an important mechanism for the preservation of cell integrity and survival. By recycling cytosolic macromolecules and organelles, autophagy provides essential nutrients and the clearance of cellular proteins [20,36]. In recent years, the role of autophagy has been discussed in relation to a spectrum of diseases, such as cancer, neurodegenerative, autoimmune and cardiovascular diseases [37]. 

Autophagy occurs in the nucleated cells of an organism. The process of autophagy in platelets is an important regulator of intra-vascular NETs formation and thrombosis [17]. Ouseph, et al. demonstrated that the process of autophagy not only occurs when platelets are at rest, but also during their activation. A deficient autophagy can produce unidentified platelet dysfunction [38]. 

In regard to autoimmune processes, the function of autophagy as a promotor of the survival of cells resistant to apoptosis is a current topic of investigation. Amaravadi et al. postulate that autophagy can be an adaptive mechanism that contributes to cell survival and resistance to therapy-induced apoptosis in a Myc-induced model of lymphoma [39]. Likewise, disbalance in immunologic-related function, such as the removal of intracellular pathogens, secretory pathways (including vesicle trafficking), autophagic regulation of ROS, pro-inflammatory signaling and antigen presentation, often trigger autoimmunity [40].

Cytokines play an important role in the regulation of autophagy. The processing and secretion of IL-1b, IL-18 and IL-1a by macrophages and dendritic cells are negatively regulated by autophagy. Conversely, autophagy positively regulates the transcription and secretion of TNF-α, IL-8 and, possibly, IL-6 and type I IFN [41]. Toll-like receptors (TLR) and NOD-like receptors (NLR) are potent inducers of autophagy due to their ability to recognize different pathogens, stress factors and cytokines [40,42].

### 2.2. NETs in Antiphospholipid Syndrome and Thrombosis

Antiphospholipid syndrome (APS) is an autoimmune disease characterized by the presence of elevated titers of antiphospholipid antibodies (aPL). These antibodies are predisposed to arterial and venous thrombosis and fetal loss [43].

One of the dominating autoantibodies in this syndrome targets beta-2-glycoprotein 1 (B2GP1), a circulating phospholipid-binding glycoprotein, secreted by the liver, monocytes, trophoblasts, endothelial cells and platelets [44]. The presence of anti-B2GP1 is frequently associated with thrombotic events, pro-atherogenic mechanisms and vascular cell dysfunction [45].

The definition of APS, according to the Sidney Classification Criteria, states that there must be clinical evidence of vascular thrombosis and/or pregnancy-related morbidity and one of the following laboratory criteria: anticardiolipin antibodies, anti-B2GP1 antibodies or lupus anticoagulant. Furthermore, in order to be classified as APS, there should be at least 12 weeks, and no more than five years, between the clinical manifestation and the positive aPL test [46].

Actually, there is no targeted treatment for APS, and current therapies focus on the management of thrombosis with long-term anticoagulant medication [47]. The mechanisms by which antiphospholipid antibodies induce thrombosis are still unclear. 

Neutrophils have been observed to be significantly related to arterial and venous thrombosis. During the autoimmune process, NETs components can be recognized by the immune system as an autoantigen that directly or indirectly influence the pathogenesis of a variety of inflammatory and autoimmune diseases.

In recent years, studies on NETs have revealed evidence that autoantibodies against B2GP1 induce NETs and enhance thrombosis. Yalavarthi, et al. [48] described the release of NETs, promoted by anticardiolipin antibodies, as a new possible mechanism of thrombosis in antiphospholipid syndrome. Confirming the hypothesis that antiphospholipid antibodies activate neutrophils to release NETs, the investigators demonstrated that isolated neutrophils of the patients with APS enhanced spontaneous NETs release, when compared with controls. In addition, a positive correlation between anti-B2GP1 IgG, lupus anticoagulant, anticardiolipin IgG and circulating MPO-DNA complexes was found, showing a correlation between the level of circulating MPO-DNA complexes and NETs in vivo. However, no correlation was observed between MPO-DNA and anti-cardiolipin antibodies IgM and IgA. A significant statistical difference was confirmed between “triple-positive” patients for lupus anticoagulant, anti-B2GP1 IgG and anti-cardiolipin IgG antibodies and “single-positive” patients and their subsequent correlation with MPO-DNA levels. The stimulation of neutrophils with isolated total IgG fractions from “triple-positive” patients with APS produces significant NETs release when compared with healthy controls. After the depletion of the anti-B2GP1 IgG fraction, the NETs abrogate. By utilizing different laboratory methods, B2GP1 was detected on the neutrophils’ surface. This discovery can explain the binding of anti-B2GP1 antibodies with neutrophils and the consequent triggering of NETosis. Another interesting observation was that both ROS formation and TLR4 engagement were required for aPL-mediated NETs release. In contrast, PMA-stimulated NETosis was TLR4-independent. These data enable one to consider the TLR4 as a possible mediator of aPL stimulation in neutrophils. 

In a recently published study, Meng et al. demonstrated, through mice models in vivo, that the administration of IgG in APS patients had a prothrombotic effect. Moreover, APS thrombi were enriched in NETs. Thus, the stimulation of mouse neutrophils by APS IgG resulted in NETosis. In addition, this group of researchers showed that both neutrophil depletion and DNase administration have been seen to abrogate thrombosis in APS mice [49].

While aPL/neutrophil interplay in obstetric APS is still unknown and further investigation is required, a number of studies suggest a pathogenic role of NETs in aPL-negative patients experiencing pre-eclampsia [50].

Leffler et al. proved that patients with systemic lupus erythematosus (SLE) have a defect in DNase-mediated NETs degradation [51]. Nevertheless, this phenomenon is not significant in patients with APS; and if present, does not correlate with the presence of aPL antibodies, such as anti-B2GP1, anti-cardiolipins or lupus anticoagulant. There is no evidence that aPL antibodies coincide with or cause failed NETs degradation [52]. 

NETs contribute both to arterial and venous thrombosis through the following mechanisms: its ability to bind and activate platelets, tissue factor (TF) and coagulation factor VII, which accelerate the thrombus formation [38]. 

Kambas et al. focused on the role of neutrophils in the coordination between inflammation and coagulation. The researchers demonstrated that TF-bearing NETs released from the neutrophils of patients with sepsis play a key role in the activation of the coagulation system by triggering thrombin generation. Furthermore, it was shown that the autophagy-dependent mechanism is involved in the extracellular localization of TF in NETs [53,54]. In another study, this group of investigators propose that TF expressed by NETs, as well as the TF expressed by microparticles could be the trigger of a new mechanism for the induction of inflammation and thrombosis in active ANCA-associated vasculitis [55].

In vitro and in vivo studies have shown that NETs contribute to thrombus formation and coagulation factors involved in clotting [56,57] through a variety of components: high amounts of TF expressed by NETs at sites of inflammation produce localized activation of the coagulation cascade; the DNA component of NETs activates factor XII, initiating contact pathway coagulation, leading to fibrin formation; histones, components of extracellular nucleosomes in NETs, activate platelets and sequester certain anticoagulant molecules like thrombomodulin and protein C. In addition, neutrophil serine proteases (neutrophil elastase and cathepsin G), present in NETs, generate degradation and inactivation of the anticoagulant molecule tissue factor pathway inhibitor (TFPI). Finally, NETs suppress fibrinolysis by intercalating into the fibrin clots [5,18,22,58,59,60].

Additional information on how these mechanisms secure the release of NETs is necessary in order to better understand the physiological conditions of neutrophils’ function. The unique link between inflammation and thrombosis is extracellular DNA. When tested, it was discovered that markers of extracellular DNA traps are abundant in deep venous thrombosis (DVT) [5].

Maternal TF on neutrophils is a necessary trigger in the pathogenesis of APS, which results in fetal loss. This demonstrates an important connection between complement components, TF and neutrophils [61].

The significant role of TF in thrombosis is based on vascular injury by factor VIIa binding. Furthermore, it has been established that TF is important in thrombosis and inflammation in APS patients [62]. Ritis, et al. observed that the neutrophils of healthy individuals stimulated with APS serum are able to express TF [63]. Moreover, the interaction of complement with neutrophils produces the generation of TF-dependent coagulation activity and the induction of TF-dependent thrombosis. This interaction occurs through C5a, a potent chemotactic factor, which is activated through C5aR receptors expressed on their surfaces. After activation, neutrophils migrate to inflamed tissues, infiltrating the injured sites [61]. 

**Figure 1:**
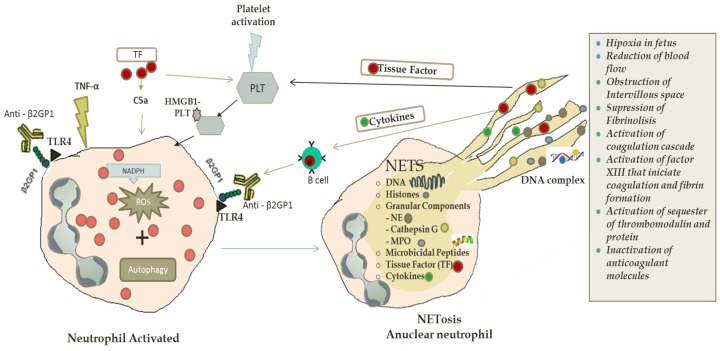
Trigger factors, such as activated platelets through the HMGB1-platelet complex, pro-inflammatory cytokines (TNF-α), tissue factor (TF) or the interaction of anti-B2GP1 with surface B2GP1 in antiphospholipid syndrome via TLR4, prompt NETosis. ROS by NADPH and autophagy induce NETs formation process. During NETosis, the components of NETs (DNA complex, histones, microbicidal peptides, cytokines, granular components) are released. If this autoimmune vicious cycle occurs, TF produced by NETs also activates platelets, as well as cytokines from NETs participation in the activation of B cells to produce autoantibodies. ROS: reactive oxygen species; NADPH: nicotinamide adenine dinucleotide phosphate; PLT: platelets; HMGB1: high mobility group box 1; TNF-α: tumor necrosis factor α; Anti-Β2GP1: anti-beta 2 glycoprotein 1; B2GP1: beta 2 glycoprotein 1; TLR4: Toll-like receptor 4; NE: neutrophil elastase; MPO: myeloperoxidase; DNA: deoxyribonucleic acid; TF: tissue factor.

Increasing evidence shows that neutrophils are related to obstetric antiphospholipid syndrome, in which pathogenic NETosis is initiated by aPL binding trophoblasts. This binding produces the activation of complement cascade leading to C5a generation. The involvement of C5a with a C5a receptor on neutrophils produces the TF expression. The TF expression increases cellular activation (ROS production), leading to inflammation, injury and fetal death [64]. (See Figure 1).

## 3. New Mechanisms of Old Therapeutics Agents 

Evidence of NETs formation and its relationship with thrombosis has led to the increased investigation of new mechanisms of action and the existent drugs.

### 3.1. Acetylsalicylic Acid 

Acetylsalicylic acid (ASA) in low dose has been widely used as a therapy for obstetric APS due to its antiplatelet mechanism of action by the inhibition of platelet cyclooxygenase [65]. Lapponi et al. demonstrated that ASA and nuclear factor NF-kB inhibitors significantly decrease NETs generation from neutrophils stimulated with phorbol 12-myristate 13-acetate (PMA) or TNF-α; while dexamethasone has no such effect [66]. 

### 3.2. Heparins

Heparins, a mixture of multifunction glycosaminoglycans, are principal drugs in the treatment of thrombosis and thromboprophylaxis in high-risk patients with obstetric APS. These drugs have both antithrombin-dependent and antithrombin independent activities. Heparins have the ability to almost completely dismantle NETs through the destabilization of backbone formed by chromatin fibers. In addition, they remove platelet aggregations and releases histones from chromatin, interfering with neutrophil-platelet cross-talk [67]. 

The capacity of heparin to block the binding of HMGB1 to the surface of macrophages also contributes in the control of NETosis through inhibiting the induction of pro-inflammatory cytokines, including TNF-α [68]. 

Moreover, pre-treatment with low-molecular-weight heparins (LMWH) has an effect on the induction of autophagy and NETs formation in vitro and in vivo: LMWHs at a “prophylactic dose”, used for the prevention of obstetric complications related to APS, inhibit the ability of neutrophils to activate autophagy, to mobilize the granule content and to form NETs [69]. 

### 3.3. Hydroxychloroquine and Chloroquine

Hydroxychloroquine (HCQ) and chloroquine (CQ) are antimalarial immunomodulators. The antimalarials are a cornerstone in the treatment of SLE and APS. HCQ has been shown to reduce the risk of thromboembolic events in both patients with SLE and positive aPL. These drugs block the processing of NETs through TLR9 in plasmacytoid dendritic cells (pDCs) [70].

CQ significantly inhibits NETs formation in controls and lupus nephritis neutrophils in vitro [71]. CQ also plays an important role in regulating NETosis through its autophagy inhibitor property. CQ has an effect on the lysosomal degradation pathway, enhancing the autophagic vesicle clearance. HCQ, a derivative of CQ, has a similar mechanism of action [39,72].

### 3.4. Vitamin D 

The immunomodulator vitamin D calcitriol/1,25(OH)2D3 reduces the production of the mediators of the inflammation and ROS in neutrophils [73]. Handono, et al. found that vitamin D calcitriol/1,25(OH)2D3 could decrease NETosis activity and reduce endothelial damage in patients with SLE and hypovitaminosis D [74].

### 3.5. Vitamin C

Vitamin C, as an endogenous antioxidant, is essential in diseases prevention. It was discovered that vitamin C operates as a novel regulator of NETs formation in pathways associated with sepsis. An increase of vitamin C has been shown to weaken NETosis in septic mice. Furthermore, polymorphonuclear cells, deficient in vitamin C, were more susceptible to produce NETs via NFκB activation, which develop ROS production and autophagy, indispensable factors for NETs formation [75].

Vitamin C-deficient neutrophils show an increase of the expression of peptidyl arginine deiminase 4 (PAD4). Furthermore, citrullination with PAD4 produces chromatin decondensation, which is essential in NETs formation [75,76]. 

Other evidence confirms that vitamin C attenuates NETosis induced by PMA in neutrophils from healthy volunteers [75]. 

However, various randomized studies could not demonstrate the effectiveness of vitamin C supplementation in preventing cardiovascular events, including stroke [77,78,79].

## 4. Biologic Anti-Cytokine Therapy

As previously discussed, cytokines play an important role in the process of NETosis. It was demonstrated that the inhibition of TNF and IL-17 abates NETosis in patients with rheumatoid arthritis [80]. Several authors find the administration of TNF-α inhibitors (adalimumab, etanercept, infliximab) useful in the treatment of refractory obstetric antiphospholipid syndrome [81]. Nevertheless, special attention must be paid to certolizumab pegol, the PEGylated Fab’ fragment of humanized anti-TNF-α monoclonal antibody, as a potential treatment of this condition [82]; due to the fact that this TNF-α inhibitor has a minimal placental transfer, measured by cord blood levels at birth, when compared with infliximab and adalimumab [83]. 

### 4.1. Statins

The pleiotropic immunomodulatory effect, anti-inflammatory and anti-thrombotic properties of statins have interested researchers and physicians during the last few decades [84]. The ability of statins to downregulate tissue factor and other prothrombotic markers was described by several researchers [85,86,87]. Nevertheless, regarding NETosis, Chow et al. [88] demonstrated that statins enhance NETs production despite the existing evidence of its capability to reduce ROS production [89]. The results of this study suggest that statins can promote NETs formation in response to a lower threshold level of ROS signaling. 

Thus, although the boost of NETosis by statins has been shown to be useful in the treatment of sepsis and other infectious diseases, which lead to immunosuppression [90], the same effect can explain incidences of statin-related autoimmune reactions [91,92,93]. 

### 4.2. Potential Therapeutic Agents

The possibility to modulate NETosis demands more research on new therapeutic opportunities. Among molecules that have potential effect on neutrophil and NETs formation are: DNase 1 (enzymatic degradation of NETs) [52,53], eculizumab (anti-C5a monoclonal antibody, reduce neutrophil activation) [94], rituximab and belimumab (B cell depletion, downregulation of NETs formation through control of antibodies production) and Resatrovid (TAK-242, a small-molecule-specific inhibitor of Toll-like receptor 4 signaling, inhibitor of NETs release by human neutrophils) [19,95].

## 5. Conclusions

There is much evidence with respect to the participation of NETs in thrombotic events. Nevertheless, more investigation is needed to completely elucidate the role of the aPL in NETs formation, as well as its participation in the pathologic mechanisms of the APS, especially obstetric APS. Mechanisms that involve NETs in pathologic processes may differ in vivo and in vitro. Furthermore, the structure and property of NETs might vary depending on the pathological and physiological conditions. Continued research on the mechanisms of action of current market drugs, as well as the advancing development of new medication, will evolve treatments for patients diagnosed with different forms of APS.
